# A Sequence-Independent Strategy for Amplification and Characterisation of Episomal Badnavirus Sequences Reveals Three Previously Uncharacterised Yam Badnaviruses

**DOI:** 10.3390/v8070188

**Published:** 2016-07-07

**Authors:** Moritz Bömer, Aliyu A. Turaki, Gonçalo Silva, P. Lava Kumar, Susan E. Seal

**Affiliations:** 1 Natural Resources Institute, University of Greenwich, Central Avenue, Chatham, Kent ME4 4TB, UK; a.turaki@agshare.today (A.A.T.); G.Silva@greenwich.ac.uk (G.S.); S.E.Seal@greenwich.ac.uk (S.E.S.); 2 International Institute of Tropical Agriculture (IITA), Oyo Road, PMB 5320, Ibadan, Nigeria; L.Kumar@cgiar.org

**Keywords:** yam, *Dioscorea* spp., badnavirus, endogenous pararetrovirus, episomal badnavirus, diagnostics, rolling circle amplification, Sub-Saharan Africa

## Abstract

Yam (*Dioscorea* spp.) plants are potentially hosts to a diverse range of badnavirus species (genus *Badnavirus*, family *Caulimoviridae*), but their detection is complicated by the existence of integrated badnavirus sequences in some yam genomes. To date, only two badnavirus genomes have been characterised, namely, *Dioscorea bacilliform AL virus* (DBALV) and *Dioscorea bacilliform SN virus* (DBSNV). A further 10 tentative species in yam have been described based on their partial reverse transcriptase (RT)-ribonuclease H (RNaseH) sequences, generically referred to here as *Dioscorea* bacilliform viruses (DBVs). Further characterisation of DBV species is necessary to determine which represent episomal viruses and which are only present as integrated badnavirus sequences in some yam genomes. In this study, a sequence-independent multiply-primed rolling circle amplification (RCA) method was evaluated for selective amplification of episomal DBV genomes. This resulted in the identification and characterisation of nine complete genomic sequences (7.4–7.7 kbp) of existing and previously undescribed DBV phylogenetic groups from *Dioscorea alata* and *Dioscorea rotundata* accessions. These new yam badnavirus genomes expand our understanding of the diversity and genomic organisation of DBVs, and assist the development of improved diagnostic tools. Our findings also suggest that mixed badnavirus infections occur relatively often in West African yam germplasm.

## 1. Introduction

Yam (*Dioscorea* spp.) is a major staple food, which plays an important role in food security and income generation for the growing population of Sub-Saharan Africa (SSA) [1]. Virus accumulation in the yam’s vegetatively propagated germplasm currently decreases production and hinders international exchange of germplasm [2,3]. In particular, *Dioscorea* bacilliform viruses (DBVs) are widespread viruses that have been detected in all the major yam species cultivated worldwide [2,4,5,6,7,8,9,10,11,12,13]. Under field conditions, DBVs are spread primarily via tubers used as planting material, but are also known to be transmitted naturally by several species of mealybugs (family *Pseudococcidae*) in a semi-persistent manner [5,14,15,16]. DBV infections in yams can cause leaf veinal chlorosis, necrosis and distortions (e.g., puckering and crinkling), but may also be symptomless [3]. The extreme heterogeneity of badnaviruses, together with the problem of yam being an under-researched crop, means that knowledge of this class of viruses in this crop is presently insufficient to assess the impact it has on crop yields and to develop more reliable diagnostic tools.

DBVs belong to the genus *Badnavirus* of the family *Caulimoviridae* [17]. Badnavirus genomes are made of a single molecule of non-covalently closed circular double-stranded (ds) DNA of ~7.2–9.2 kbp, and virions are bacilliform (30 × 130 nm) [17]. Badnaviruses contain three open reading frames (ORFs) on the positive DNA strand of their genome with each strand having a single discontinuity [17,18,19,20]. Since the first report of a DBV in *D. alata* from the Caribbean in the 1970s [21,22], several hundred partial reverse transcriptase (RT)-ribonuclease H (RNaseH) sequences have been characterised [2,8,9,11,12,23,24]. Analyses of these sequences led to the proposition of 12 badnavirus species in *Dioscorea* spp. according to the International Committee on Taxonomy of Viruses (ICTV) demarcation criteria of species differing by >20% in their partial RT-RNaseH sequences [2,9,17]. However, complete genome sequences of only two species of DBVs are available in GenBank: *Dioscorea bacilliform AL virus* (DBALV) from *D. alata* of Nigerian origin, with a genome size of ~7.4 kbp [4], and *Dioscorea bacilliform SN virus* (DBSNV) from *D. sansibarensis* of Benin origin, with a genome size of ~7.3 kbp [6]. These sequences represent the only recognised yam badnavirus species by current taxonomic criteria [17]. There is a need to characterise more yam badnaviruses in order to develop improved diagnostic tests to enable the international exchange of yam germplasm and determine virus epidemiology and the impact of specific badnaviruses on yam yields.

The use of molecular diagnostic tools involving serological and nucleic acid-based techniques for DBV detection are subject to several complications. High genomic heterogeneity of yam badnaviruses [2,3,9] poses a significant challenge for the development of primers that detect all virus strains and hence reliable diagnostic tools, as also experienced for badnaviruses infecting other crops such as banana and sugarcane [25,26,27,28,29]. The discovery of integrated badnavirus sequences in the genomes of Guinea yam (*Dioscorea cayenensis-rotundata* complex) host plants [3,30] further complicates detection of true (episomal) badnavirus infections by PCR, as experienced previously for detection of banana streak viruses (BSVs) [31,32]. Integrated badnavirus sequences, hereafter termed endogenous badnavirus sequences, are considered to have been the cause for almost all yam plants of the *D. cayenensis-rotundata* complex testing positive for badnavirus by PCR [3,7,9].

Three badnavirus detection techniques have been proposed to overcome the problem of differentiating endogenous from exogenous badnaviral DNA, namely immunocapture-PCR (IC-PCR), reverse transcription (RT)-PCR and rolling circle amplification (RCA) [31,33,34]. IC-PCR has been developed for the detection of the episomal form of BSV, removing positive PCR results generated from an integrated counterpart in *Musa* genomes [31]. Likewise RT-PCR has also been employed to distinguish between integrated and episomal virus in fig accessions [34]. Although IC-PCR has subsequently been reported as a standard method for indexing BSV [32,35,36], the method does have limitations for many badnaviruses due to a lack of antisera able to capture all badnavirus isolates [32,37]. For yam badnaviruses, no specific antisera has been reported to date [2,3], making IC-PCR unsuitable as a diagnostic tool for DBV detection.

RCA is a sequence-independent approach that is often used in the laboratory for the amplification of circular DNA virus genomes, thus overcoming the shortcoming of primer dependency in PCR for the amplification of viruses with a circular DNA genome [33,38,39]. The RCA technique has been used successfully for the study of several other circular DNA viruses such as sweet potato leaf curl virus [40] and beet curly top virus [41] of the family *Geminiviridae*, and BSV [39,42] and fig badnavirus 1 [34] of the family *Caulimoviridae.* It was proposed that RCA could be used for the specific detection of episomal forms of yam badnaviruses [30]. Hence the aim of this study was to evaluate the suitability of RCA for DBV diagnostics with the by-product of characterising novel episomal badnaviruses infecting *Dioscorea* spp. RCA was primarily chosen as this method effectively differentiated circular viral DNA from linear integrated sequences for selective amplification of episomal DNA, as illustrated for BSV [33]. The selectiveness of RCA targeting circular viral DNA over linear integrated sequences and by that overcoming the problems associated with endogenous sequences in virus indexing is discussed in this study.

Here, we use a combination of RCA and PCR for the amplification and characterisation of nine complete genomic yam badnavirus sequences corresponding to existing and previously undescribed yam badnavirus species. Based on nucleotide sequence analysis of the RT-RNaseH-coding region of open reading frame 3, we propose that six sequences isolated from different *D. rotundata* accessions be recognised as two new species and be designated as Dioscorea bacilliform RT virus 1 (DBRTV1, GenBank KX008574; DBRTV1-[2RT], GenBank KX008575; DBRTV1-[3RT], GenBank KX008576) and Dioscorea bacilliform RT virus 2 (DBRTV2, GenBank KX008577; DBRTV2-[2RT], GenBank KX008578; DBRTV2-[3RT], GenBank KX008579). Two further sequences from *D. alata* and *D. rotundata* can be recognised as additional isolates of DBALV and be called DBALV-[2AL] (DBALV-[2ALa], GenBank KX008571 and DBALV-[2ALb], GenBank KX008572) and DBALV-[3RT] (GenBank KX008573), respectively.

## 2. Materials and Methods

### 2.1. Plant Material

Yam breeding lines and landraces (61 samples in total) of *D. alata* (*n* = 15), *D. bulbifera* (*n* = 6), *D. cayenensis* (*n* = 3), *D. dumetorum* (*n* = 5), *D. esculenta* (*n* = 5) and *D. rotundata* (*n* = 27) used in this study were provided by the International Institute of Tropical Agriculture (IITA, Ibadan, Nigeria). Tubers were grown in a quarantine aphid-proof glasshouse at the Natural Resources Institute (NRI, Chatham, UK), as described by Mumford and Seal [43]. Selected individual leaf samples were collected from each plant in small polythene bags (10 cm × 15 cm) and processed immediately.

### 2.2. Total Nucleic Acid Extractions from Yam Leaves and PCR Amplification of Badnavirus Sequences

Total nucleic acids were extracted from fresh yam leaf tissue (~300 mg) using a modified CTAB method, as described by Kenyon et al. [2]. For optimal PCR and RCA detection, DNA pellets were resuspended in 2 mL resuspension buffer (50 mM Tris-Cl, 0.7 M NaCl, 10 mM EDTA, pH 7), followed by purification through Tip-100G columns (Qiagen, Manchester, UK) according to the manufacturer’s instructions. Inclusion of this column purification step was found to increase the efficiency of RCA. The final pellets were resuspended in 200 µL sterile distilled deionised water (SDW). Screening of the total nucleic acids for the presence of badnavirus sequences was performed by PCR using the generic badnavirus primer pair Badna-FP/-RP [44], amplifying a 579 bp region (528 bp excluding primer sequences and representing only complete amino acids) of the RT-RNaseH gene used for taxonomic assessment of badnaviruses [17]. To confirm the suitability of DNA for PCR amplification, all DNA samples were first screened using primers targeting the yam actin gene, as described by Silva et al. [45]. PCR amplifications were set up in 20 µL reactions containing 20 ng template, 0.5 µM of each primer, 0.25 mM of each dNTP, 1 U DreamTaq DNA polymerase and 1× DreamTaq Green buffer (Thermo Scientific, Loughborough, UK) containing 2 mM MgCl_2_. The cycle conditions for PCR amplification were 95 °C for 5 min, followed by 30 cycles of 94 °C for 20 s, 56 °C for 30 s, 72 °C for 30 s and a final extension of 72 °C for 7 min. PCR products were analysed by gel electrophoresis through 1.5% (*w*/*v*) agarose gels including 1× RedSafe nucleic acid stain (iNtRON Biotechnology, Seongnam, South Korea) in 0.5× Tris-Boric acid-EDTA (TBE) buffer. Samples that were positive using the primer pair Badna-FP/-RP were used for RCA analysis.

### 2.3. RCA, Restriction Digestion Analysis, Cloning and Sequencing

Badnavirus DNA was amplified from yam DNA extracts using an Illustra^TM^ TempliPhi 100 Amplification kit (GE Healthcare, Little Chalfont, UK), according to the manufacturer’s instructions with an additional step including the Badna-FP/-RP primer pair, as described in James et al. [33]. The RCA reactions were incubated at 30 °C for 18 h, followed by enzyme deactivation at 65 °C for 10 min. The RCA products were digested using restriction enzymes, selected based on the restriction profile of DBSNV (DQ822073) identified in silico using software NEBcutter V2.0 [46]. The enzymes that generated useful restriction profiles were *Hin*dIII (5′-AA/GCTT-3′) and *Pst*I (5′CT/GCAG-3′), as well as putative single cutting enzymes *Kpn*I (5′GGTAC/C-3′) and *Sph*I (5′-GCAT/GC-3′). The RCA reaction products (2 µL) were digested using 10 U of restriction enzyme according to the manufacturer’s instructions (NEB, Hitchin, UK), and electrophoresed through 0.6% (*w*/*v*) agarose gels prepared in a 0.5× TBE buffer. Bands of interest were excised under minimal UV exposure and purified using a gel extraction kit (Qiagen). Prior to ligation, the purified RCA restriction fragments were tested for their origin from badnavirus sequences by PCR using Badna-FP/-RP primers. Positive Badna PCRs were purified, cloned and sequenced, and the gained sequences were coded as follows: the first two capital letters (NG) stand for the country of origin (Nigeria) and the small letter (b or l) represents sample type (breeding line or landrace yam, respectively), the numbers following this denote the last four numbers of the corresponding IITA plant accession number and the last letters refer to the *Dioscorea* host species.

The RCA restriction fragments of badnaviruses were ligated into appropriately digested alkaline phosphatase-treated pGEM^®^-3Zf (+)-vector (Promega, Southampton, UK) or pUC19-vector (NEB) and subsequently transformed into *E. coli* JM109 competent cells (Promega) or DH5-α competent *E. coli* C29871 (NEB), according to the manufacturer’s instructions. Clones containing the appropriate insert size were selected by PCR using either SP6/T7 or M13F/M13R primer sets and subsequently grown in 5 mL overnight cultures. Plasmid DNA was extracted using GeneJET Plasmid Miniprep Kit (Thermo Scientific) according to the manufacturer’s instructions and the presence of the cloned fragments was confirmed by restriction digestion prior to sequencing. Following RCA and sequencing analysis, the gaps in the full genomes of DBRTV1-[2RT] and DBALV-[3RT] were covered by PCR using outfacing primers designed based on the RCA-generated sequences of the respective genome sequence (Table S1). At least three independent PCR amplifications were performed and the products were directly sequenced, or sequenced after cloning in TOPO^®^ XL (Invitrogen, Life Technologies, Paisley, UK). The DBRTV1-[3RT] genome sequence was an exception as it was determined entirely based on PCR amplification using sequence-specific primers designed based on DBRTV1 and DBRTV1-[2RT] (Table S1). Polymerase chain reactions and cycle conditions were as described before but using 50 µL reactions, 2 U DreamTaq DNA polymerase, an annealing temperature of 58 °C and an extension time of up to 5 min, depending on the expected amplicon size (1 kb/min). All sequencing was performed by Source BioScience sequencing service (Nottingham, UK) using specific primers designed to walk the genome. All the primers described in this study were synthesised using Sigma oligo service (Sigma-Aldrich, Gillingham, UK) and reSource PCR purification kits (Source BioScience, Nottingham, UK) were used for purification of PCR products prior to sequencing and cloning.

### 2.4. Sequence Analysis and Phylogeny

Nucleotide sequences generated from plasmid clones and PCR products were analysed and assembled using MEGA version 6.0 [47]. In all cases, a minimum of three independent clones were sequenced in both directions in order to determine an accurate sequence of the putative full-length badnavirus genomes. Vector sequences were removed using National Centre for Biotechnology Information (NCBI) VecScreen (http://www.ncbi.nlm.nih.gov/tools/vecscreen/) and the NCBI basic local alignment search tool (BLAST). Genome maps were generated using SnapGene^®^ Viewer version 2.7.3 (from GSL Biotech; available at snapgene.com). The edited sequences were used for similarity BLAST searches in the NCBI GenBank databases (http://www.ncbi.nlm.nih.gov/genbank/). Multiple alignments of the partial RT-RNaseH sequences were performed using the CLUSTALW default settings in MEGA version 6. Complete badnavirus genomes were aligned and a percent similarity matrix was generated using Multiple Alignment using Fast Fourier Transform (MAFFT; http://www.ebi.ac.uk/Tools/msa/mafft/) [48]. Protein sequences were aligned using CLUSTAL OMEGA (http://www.ebi.ac.uk/Tools/msa/clustalo/) [49]. Following pair-wise comparisons of nucleotide sequences and their deduced amino acid sequences, phylogenetic relationships were analysed by MEGA version 6 using the Maximum Likelihood method with the Kimura 2-parameter model [50] for the complete genomic sequences and the Poisson model for the protein sequences. The robustness of the trees was determined by generating bootstrap consensus trees using 1000 replicates. Full-length genome sequences were assembled in MEGA version 6 and the identification of putative open reading frames (ORFs) was done using the NCBI ORF finder (http://www.ncbi.nlm.nih.gov/gorf/gorf.html). Conserved domains of the putative gene products were searched using the NCBI conserved domain tool (http://www.ncbi.nlm.nih.gov/Structure/cdd/wrpsb.cgi). The following virus genome sequences obtained from the GenBank were used for comparative analyses: Banana streak virus Acuminata Yunnan (BSAcYNV, NC_008018); Banana streak CA virus (BSCAV, NC_015506); *Banana streak GF virus* (BSGFV, NC_007002); *Banana streak IM virus* (BSIMV, NC_015507); *Banana streak MY virus* (BSMYV, NC_006955); *Banana streak OL virus* (BSOLV, NC_003381); *Banana streak UA virus* (BSUAV, NC_015502); *Banana streak UI virus* (BSUIV, NC_015503); *Banana streak UL virus* (BSULV, NC_015504); *Banana streak UM virus* (BSUMV, NC_015505); *Banana streak VN virus* (BSVNV, NC_007003); *Bougainvillea chlorotic vein-banding virus* (BSCVBV, NC_011592); *Cacao swollen shoot virus* (CSSV, NC_001574); *Citrus yellow mosaic virus* (CiYMV, NC_003382); *Commelina yellow mottle virus* (ComYMV, NC_001343); Cycad leaf necrosis virus (CyLNV, NC_011097); *Dioscorea bacilliform AL virus* (DBALV; X94576–X94581); *Dioscorea bacilliform SN virus* (DBSNV, DQ822073); Dracaena mottle virus (DrMV, NC_008034); *Fig badnavirus 1* (FiBV-1, NC_017830); *Gooseberry vein banding associated virus* (GVBV, NC_018105); *Grapevine vein clearing virus* (GVCV, NC_015784); *Kalanchoë top-spotting virus* (KTSV, NC_004540); *Rice tungro bacilliform virus* (RTBV, NC_001914); Pelargonium vein banding virus (PVBV, NC_013262); *Pagoda yellow mosaic associated virus* (PYMAV, KJ013302); *Pineapple bacilliform CO virus* (PBCV, NC_014648); *Piper yellow mottle virus* (PYMV, NC_022365); *Sugarcane bacilliform IM virus* (SCBIMV, NC_003031); *Sugarcane bacilliform MO virus* (SCBMOV, NC_008017); *Sugarcane bacilliform virus* (SCBV, NC_013455); *Taro bacilliform virus* (TaBV, NC_004450).

## 3. Results

### 3.1. Rolling Circle Amplification Combined with Restriction Fragment Length Polymorphism (RCA/RFLP)

**Figure 1:**
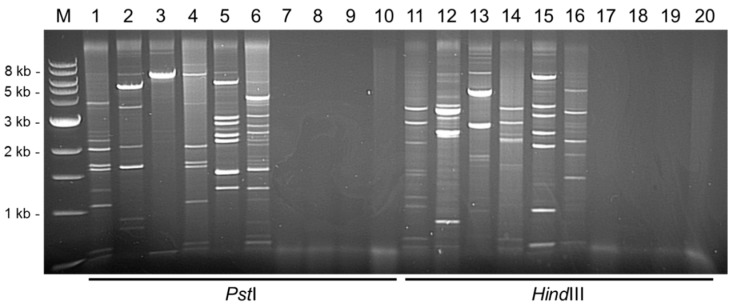
Restriction enzyme analysis of RCA products from DNAs of 10 *Dioscorea* species digested with *Pst*I (lane 1–10) or *Hin*dIII (lane 11–20) restriction enzymes analysed on 0.6% (*w*/*v*) agarose gel. Differing restriction digestion patterns suggest the presence of different episomal badnavirus sequences. M = 1 kb DNA ladder (NEB), lane 1 and 11 = TDr 89/02475, lane 2 and 12 = TDa 00/00005, lane 3 and 13 = TDa 85/00250, lane 4 and 14 = TDr 95/18544, lane 5 and 15 = TDa 95/00310, lane 6 and 16 = TDr 1892, lane 7 and 17 = TDa 01/00039, lane 8 and 18 = TDr 98/01166, lane 9 and 19 = TDa 89/02677 and lane 10 and 20 = TDa 96/00629.

To evaluate the diagnostic potential of RCA in DBV detection, a total of 61 yam DNAs that scored positive for badnavirus sequences using Badna-FP/-RP primers were screened by RCA. Analysis of RCA-positive products by restriction digestion using *Hin*dIII and *Pst*I revealed a range of restriction patterns when screening a selection of yam accessions (Figure 1). Twenty-six DNAs (43%) generated clear RCA bands, with the remaining samples being either RCA-negative or resulting in very faint bands that were hard to differentiate clearly from background smearing. Samples that showed no restriction fragments are illustrated in Figure 1 (lanes 7–10 *Pst*I-digested and lanes 17–20 *Hin*dIII-digested), and were considered to be due to absence of episomal circular DNA or potential inhibition of the RCA assay due to plant compounds present in the DNA extractions.

RCA restriction fragments of high intensity were excised from positive samples, purified and screened for the presence of the RT-RNaseH coding region of the genus *Badnavirus* before cloning. Problems were experienced in relation to the excision of bands in close proximity to other bands, and the cloning of bands of low intensity. For 10 RCA-positive samples, RCA restriction fragments were amplified by PCR using Badna-FP/-RP primers and directly sequenced. Where a generated sequence indicated the presence of an unknown badnavirus species, attempts were made to clone all RCA restriction fragments of that given sample. Sequences typical of yam badnaviruses were obtained for the majority of successfully cloned RCA restriction fragments (Table 1), although yam plastid DNA sequences were also identified in a few clones (98%–99% sequence identity to *Dioscorea rotundata* plastid, complete genome, GenBank KJ490011.1 [51]).

The objective of this study was to amplify full-length episomal badnavirus genomes. Sample TDa 85/00250 upon *Pst*I-digestion gave rise to a large band of approximately 7.1 kbp (presumably representing a near to full-length DBV genome), and a second band around 430 bp (Figure 1, lane 3). The presence of an episomal badnavirus in this sample was supported by the sum of the two *Hin*dIII-digestion bands (~4.8 and ~2.6 kbp) (Figure 1, lane 13) being ~7.4 kbp which is of the expected approximate size of a complete badnavirus genome. Two sequences of the complete badnavirus genome, named DBALV-[2ALa] and DBALV-[2ALb], with 99.7% nucleotide identity to each other, were assembled from the excision and cloning of these 7.1 kbp and ~430 bp fragments in Figure 1 lane 3. The DBALV-[2ALa/b] genomes obtained confirmed the RCA restriction fragments gained after *Hin*dIII digestion with an additional band of 184 bp (Figure S3).

In all other lanes containing RCA products, the sum of all bands per lane exceeded the typical size of a badnavirus genome (7–9 kbp). These findings suggested that mixed infections were occurring, which was confirmed by the amplification of multiple badnavirus isolates from single samples, as evidenced by their partial RT-RNaseH sequences (Table 1). In total, mixed infections were observed in six of the 12 samples studied in detail by RCA (Table 1). Double infections were present in TDa 00/00005, TDr 89/02475 and TDr 95/18544, whereas triple infections were identified in TDa 95/00310, TDr 1892 and TDr 1892B (Table 1). The detection and characterisation of DBRTV1-[2RT], DBRTV2-[3RT] and DBALV-[3RT] full-length genomes confirmed the presence of three badnavirus isolates in TDr 1892B. In TDa 95/00310 RCA restriction fragments also indicated a triple infection, with bands adding up to ~21.5–23 kbp following *Pst*I and *Hin*dIII digestion (Figure 1, lane 5 and 15). However, the estimation of number of isolates by the sum of all RCA fragments is far from reliable due to the potential amplification of plant DNA by RCA, or due to incomplete restriction digestion. This is illustrated by sample TDa 00/00005 (Figure 1, lane 12), where bands add up to greater than 17 kbp. Although this suggested the presence of more than two episomal badnaviruses, PCR amplification and sequencing of the fragments revealed the presence of only two badnavirus sequences with the remainder of the bands originating from yam plastid and mitochondrial circular DNA.

Similar restriction patterns were observed across samples and suggest the presence of similar strains of badnaviruses. Such a situation was, for example, presented in *D. rotundata* sample TDr 95/18544 (Figure 1, lane 4) from which sequences of two badnaviruses were generated that were also found individually in *D. rotundata* TDr 89/02475 (Figure 1, lane 1) and *D. alata* TDa 85/00250 (Figure 1, lane 3). Figure 1 lane 1 and 4 show identical bands of around 2.1, 1.7, 1.6, 1.1, 0.6 and 0.5 kbp observed following *Pst*I digestion, and 3 and 3.5 kbp *Hin*dIII-digested products in the corresponding lanes 11 and 14. All six *Pst*I-digested fragments from accession TDr 89/02475 (Figure 1, lane 1) were cloned and overlapping sequences were combined with *Hin*dIII-digested RCA sequence products of 3 and 3.5 kbp (Figure 1, lane 11) to form the full-length genome DBRTV1 (Table 1). Restriction sites of the DBRTV1 genome (Figure S3) confirmed the observed restriction patterns. The partial RT-RNaseH sequence NGb1844_Dr1 (Table 1) amplified by PCR from the RCA product of accession TDr 95/18544 (Figure 1, lanes 4 and 14) has 100% sequence identity to the corresponding DBRTV1 sequence of TDr 89/02475 (Figure 1, lanes 1 and 11).

An additional complete genome sequence, named DBRTV1-[3RT], with 99.8% sequence identity to DBRTV1 was generated from another sample TDr 89/02475A by overlapping PCR products amplified using primers designed on the basis of the full-length DBRTV1-[2RT] genome (Table S1). DBRTV1-[2RT] originated from a combination of overlapping RCA and PCR products from TDr 1892B (Table 1). Interestingly, DBRTV1-[3RT] was detected in the DNA extracted of TDr 89/02475A, which was grown from a tuber harvested from TDr 89/02475, the source of the DBRTV1 genome, suggesting that this episomal badnavirus strain was passed to the next generation crop through clonal propagation of the yam breeding line. The small sequence differences observed (*n* = *23) are considered to most likely be due to mutations rather than sequencing errors, as they were present in all three clones generated from independent PCR reactions.

### 3.2. Analysis of the Partial RT-RNaseH Region from Episomal RCA Sequences

**Figure 2:**
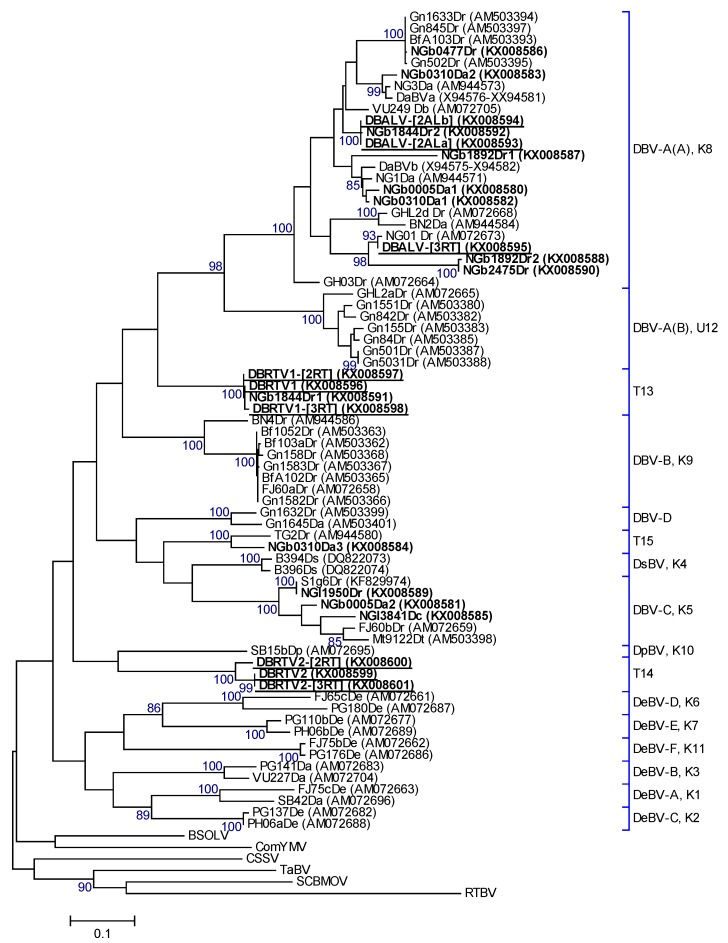
Bootstrap consensus phylogenetic tree using Maximum Likelihood method built from badnavirus 528 bp long partial RT-RNaseH nucleotide sequences of 22 yam badnavirus sequences determined in this study representing two already described and three newly proposed distinct monophyletic species groups (T13, T14, T15). Included in the analysis are partial RT-RNaseH sequences from GenBank of previously analysed yam samples by Seal et al. [3] and Eni et al. [8]. Equivalent sequences from CSSV (AJ781003), BSOLV (AJ002234), ComYMV (NC001343), SCBMOV (M89923), TaBV (AF357836) and outgroup RTBV (X57924) were added, as well as representative sequences of all monophyletic groups described by Bousalem et al. [9], where DBV-A = Dioscorea bacilliform virus A (A and B subgroups); DBV-B = Dioscorea bacilliform virus B; DBV-C = Dioscorea bacilliform virus C; DBV-D = Dioscorea bacilliform virus D; DeBV-A = Dioscorea esculenta bacilliform virus A; DeBV-B = Dioscorea esculenta bacilliform virus B; DeBV-C = Dioscorea esculenta bacilliform virus C; DeBV-D = Dioscorea esculenta bacilliform virus D; DeBV-E = Dioscorea esculenta bacilliform virus E; DeBV-F = Dioscorea esculenta bacilliform virus F; and DpBV = Dioscorea pentaphylla bacilliform virus. One monophyletic group denoted by Umber et al. [30] and 11 corresponding Kenyon et al. [2] groupings to these monophyletic groups are also given and denoted by U12 and K1–K11 respectively. Sequence names: bold = partial RT-RNaseH sequences from RCA clones, bold and underlined represent partial RT-RNaseH sequences and their GenBank accessions of the characterised full-length genomes obtained in this study. The bootstrap analysis of the sequences was 1000 replicates and the cut-off value was 85%.

Twenty-two partial RT-RNaseH sequences obtained from RCA restriction fragments and PCR amplifications were compared to sequences in GenBank, and the most similar BLAST search result of each sequence is shown in Table 1. Nucleotide pairwise comparison of these sequences with the equivalent sequence regions of the full-length DBALV and DBSNV sequences revealed 69%–96% nucleotide identities. Phylogenetic and percentage identity analyses revealed that the 22 partial RT-RNaseH sequences clustered into five putative species groups (Figure 2 and Table 1) according to ICTV species demarcation standards for the genus *Badnavirus* [17]. Two of these represent previously named species groups (K05 and K08, [2]), whereas three putative species groups identified in this study, designated as T13, T14 and T15, represent new distinct species. The partial RT-RNaseH sequences of the new species T13 and T14 did not cluster with any of the previously published sequences (Figure 2). They possess 64%–77% nucleotide identity to each other and to any previously published RT-RNaseH sequence, meeting the demarcation criteria of species differing by >20% in this region [17]. Sequence NGb0310Da3 of the new species group T15, however, has 90% nucleotide identity with TG2Dr (AM944580; [8]), available in GenBank. The latter sequence was not used in previous yam badnavirus phylogenetic analyses and species descriptions presented by Kenyon et al. [2], Bousalem et al. [9], Seal et al. [30] and Umber et al. [30].

Three badnavirus sequences, DBRTV1 (TDr 89/02475), DBRTV1-[2RT] (TDr 1892B) and DBRTV1-[3RT] (TDr 89/02475A), with 99% nucleotide identity to each other were identified within the new species group T13. These sequences share, as the closest match in GenBank, 76% nucleotide identity to the 528 bp RT-RNaseH region of the integrated sequence S2f8Dr (KF829993, [30]). The DBRTV2 (TDr 1892), DBRTV2-[2RT] (Adaka) and DBRTV2-[3RT] (TDr 1892B) complete genomes of the new species group, T14, originated from overlapping PCR products using DBRTV2 genome specific primers (Table S1). DBRTV2 and DBRTV2-[3RT] genomes are 99.7% identical and share 95% nucleotide identity with DBRTV2-[2RT]. The closest match for the partial RT-RNaseH sequences of DBRTV2 and DBRTV2-[3RT], with 72% nucleotide identity, was FJ60b_Dr (AM072659), and for DBRTV2-[2RT] the integrated sequence S1un5Dr (KF830000, [30]), also with 72% nucleotide identity (Table 1).

Several other episomal sequences were characterised from cloned RCA bands of *D. alata*, *D. cayenensis* and *D. rotundata* samples. Analyses of these sequences showed 87%–98% nucleotide identity to the ICTV-recognised yam badnaviruses, DBALV and DBSNV. Therefore, as these sequences did not represent new badnavirus species, they were not characterised further. Amino acid sequences derived from the 22 partial RT-RNaseH sequences did not possess any stop codons or frameshifts that would indicate non-functional proteins. Instead, the 22 RCA-derived sequences showed expected conserved regions, such as the “FIAVYIDDILVFS” motif at position 17–28 on the partial RT-RNaseH coding region [4,20]. Interestingly, this motif presented itself as “FVAVYIDDILVFS” in the sequences DBRTV1, DBRTV1-[2RT], DBRTV1-[3RT] and NGb1844Dr1 and as “FLAVYIDDILVFS” in the sequences DBRTV2, DBRTV2-[2RT], DBRTV2-[3RT]. The presence of this conserved motif in the C-terminal end of the RT is similar to all previous isolates of yam badnaviruses, and strongly suggests that sequences originate from members of the genus *Badnavirus*.

**Figure 3:**
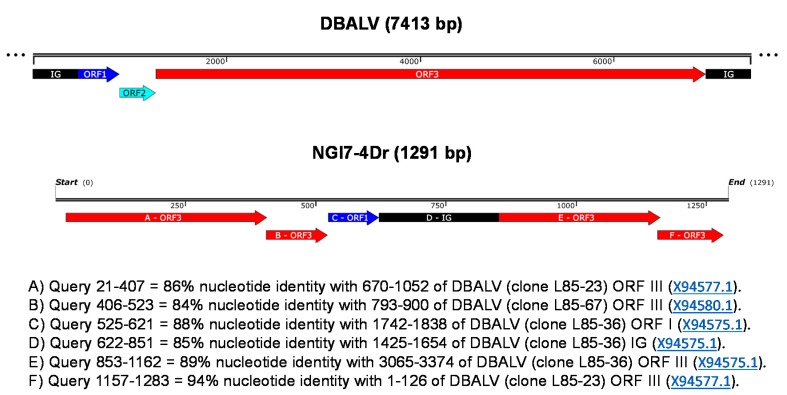
Schematic representation of the rearranged ORF3 of badnavirus clone NGl7-4Dr (KX008602) amplified from *D. rotundata* TDr 1950B by RCA. The clone length is shown in the linear scale bar and the rearranged fragments are represented in panels A-F. A non-scaled linear view of the genome organization of DBALV is shown in the top panel. The intergenic region (IG) and open reading frames (ORFs) appear with the following colour codes (adapted from Umber et al. [30]): IG, black; ORF1, dark blue; ORF2, light blue; ORF3, red.

Nucleotide and amino acid analyses of one partial badnavirus sequence obtained from a single clone (NGl7-4Dr) of an RCA product from *D. rotundata* (TDr 1950B) revealed a rearranged ORF3 sequence where the first 20 bp had no homology to badnavirus sequences (Figure 3). A sequence stretch of ORF1 also appeared inside the ORF3-rearranged sequence of this clone. The partial RT-RNaseH coding region (NGl1950Dr) obtained from a second RCA restriction fragment derived clone (NGl7-3Dr) of TDr 1950B displayed 100% nucleotide identity with endogenous Dioscorea bacilliform virus 5 (eDBV5) sequence S1g6Dr (KF829974, [30]).

### 3.3. Complete Genome Characterisations

The assembly of nine full-length viral genomes derived from four different yam accessions was achieved by cloning overlapping sequences generated following *Hin*dIII and *Pst*I digestion of RCA products, as well as PCR amplification with targeted primers (Table S1). The complete genome sequences of DBALV-[2ALa] (KX008571), DBALV-[2ALb] (KX008572), DBRTV1 (KX008574), DBRTV1-[2RT] (KX008575), DBRTV1-[3RT] (KX008576), DBRTV2 (KX008577), DBRTV2-[2RT] (KX008578), DBRTV2-[3RT] (KX008579) and DBALV-[3RT] (KX008573) have been deposited in the NCBI GenBank database. They were determined to be 7438–7708 bp in length with their GC content ranging from 42.7%–44.3% (Table S2). BLAST searches confirmed that all nine complete genomes were most similar to bacilliform viruses previously characterised from yam rather than other hosts present in the genus *Badnavirus*.

The nine genomes represented three of the five species detected in this study, namely K08, T13 and T14 (Figure 2 and Table 1). Pairwise comparison of the new T13 and T14 genomes with that of DBALV displayed 61.1%–65.4% nucleotide identity. Similarly, pairwise comparison with that of DBSNV displayed 61.7%–65.1% nucleotide identity. The other new genomes belonging to group K08 (DBALV-[2ALa/b] and DBALV-[3RT]) displayed 86.7%–90% nucleotide identity to DBALV.

Further support for the genomes being representative of the genus *Badnavirus* in the family *Caulimoviridae*, was the identification of a plant cytoplasmic initiator methionine tRNA sequence within the intergenic region at position 1–18 designating the beginning of the viral genomes [18]. The tRNA^Met^-binding site sequences of DBSNV, DBALV-[2ALa], DBALV-[2ALb], DBRTV1, DBRTV1-[2RT], DBRTV1-[3RT], DBRTV2-[2RT] (5′-TGGTATCAGAGCTTGGTT-3′) share 16 of the 18 nucleotides complementary to the consensus of plant tRNA^Met^-binding site (3′-ACCAUAGUCUCGGUCCAA-5′), whereas the in DBRTV2 and DBRTV2-[3RT] (5′-TGGTATCAGAGCTGGTGT-3′) share only 14 of these 18 nucleotides. All DBV genomes described here are identical in their first 12 nucleotides complementary to the consensus of the plant tRNA^Met^-binding site. A potential TATA-box with the sequence TATATAA located upstream of the tRNA^Met^-binding site was located, and a possible poly-adenylation signal (poly(A)-tail) was found within the intergenic region for each of the nine genomes (Table S2).

**Figure 4:**
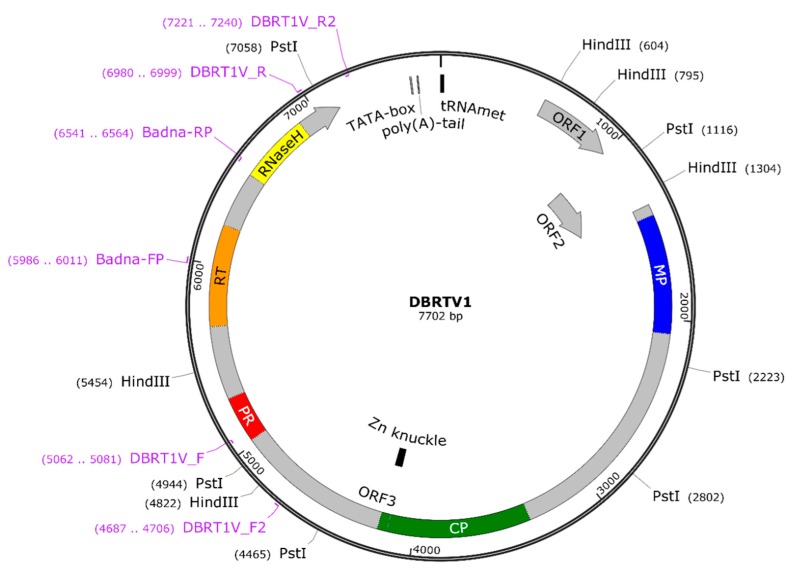
Circular representation of the DBRTV1 genome (GenBank KX008574) showing binding sites of primers (purple) used in this study and tRNA^Met^-binding site; the TATA-box; the putative poly(A)-tail; open reading frame (ORF)1; ORF2; ORF3 with putative movement protein (MP), capsid protein zinc-finger domain (CP and Zn knuckle), pepsin-like aspartate protease (PR), reverse transcriptase (RT) and RNaseH motifs; and the restriction sites for *Pst*I and *Hin*dIII.

Badnaviruses reported to date possess three conserved ORFs [19], which are arranged in tandem on the plus strand and have overlapping start and stop codons (5′-ATGA-3′) [52]. Sequence analysis using NCBI ORF finder revealed three ORFs in each of the nine genome sequences (Table S2) that are closely packed and overlap by the ATGA motif, except for ORF2 and ORF3 in DBALV-[2ALa/b] and DBALV-[3RT]. The latter differ by having a TAATG motif here, identical to DBALV [4]. In summary, the size and arrangement of these ORFs present in all nine genomes are similar to those of most badnaviruses characterised to date (Table S2). A circular representation of the DBRTV1 genome is shown as an example (Figure 4), highlighting all features typical of genomes in the genus *Badnavirus* of family *Caulimoviridae*.

### 3.4. Amino Acid Analysis and Phylogenetic Relationships

**Figure 5:**
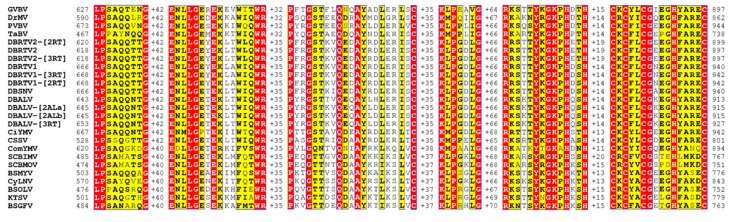
Comparison of highly conserved amino acid residues in the coat protein encoded by the N-terminal half of the ORF 3 product among DBALV-[2ALa], DBALV-[2ALb], DBRTV1, DBRTV1-[2RT], DBRTV1-[2RT], DBRTV2, DBRTV2-[2RT], DBRTV2-[3RT], DBALV-[3RT], DBALV, DBSNV, 14 badnavirus and other members of the family *Caulimoviridae* (see text for detail). Sequences were aligned with CLUSTAL OMEGA (http://www.ebi.ac.uk/Tools/msa/clustalo/) and coloured using ESPript 3.0 [57], where functional conserved amino acid residues are highlighted with yellow backgrounds and complete consistent residues with red backgrounds. Numbers of the starting and ending amino acids are specified before and after each sequence, respectively. The numbers of residues (gaps) between blocks are presented.

Multiple alignments of the putative protein sequences encoded by ORF1 and ORF2 of the nine genomes identified in this study together with the equivalent sequences of DBALV and DBSNV revealed a high degree of conserved amino acid (aa) sequences and also indicated group-specific sequence patterns (Figure S1A,B). ORF1 encodes a putative ~17 kDa (142–143 aa) protein of unknown function. ORF2 potentially encodes a ~14 kDa protein in the range of 121–126 aa. A conserved KQNN motif is found at the C-terminal region of ORF2 in other badnaviruses [53]. All nine genomes identified and DBSNV have the exact KQNN motif but in DBALV it is presented as KQYN. The polypeptide of ~214–217 kDa and 1892–1921 aa length encoded by ORF3 in the nine genomes is conserved in all badnaviruses. Using the NCBI conserved motif search, the badnavirus ORF3 encodes previously identified characteristic features including the Zinc knuckle (Zn knuckle), pepsin-like aspartate protease (PR), reverse transcriptase (RT) and ribonuclease H (RNaseH) (Figure 4 and Figure S3) [4,54,55]. The coat protein (CP) (Figure 5 and Figure S2) and movement protein (MP) (Figure 4 and Figure S3) described by Xu et al. [55] were also located. The Zn knuckle is a cysteine-rich, zinc finger-like RNA-binding domain (CXCX_2_CX_4_HX_4_C) found in the CP of all pararetroviruses [6,19,56], being located at the C-terminal end of the CP (Figure 5). The coat protein and the Zn knuckle domain within appear very highly conserved among the yam badnavirus genomes and are presented as CKCFLCG(A/E/K/N)EGH(F/Y)AREC (Figure S2).

**Figure 6:**
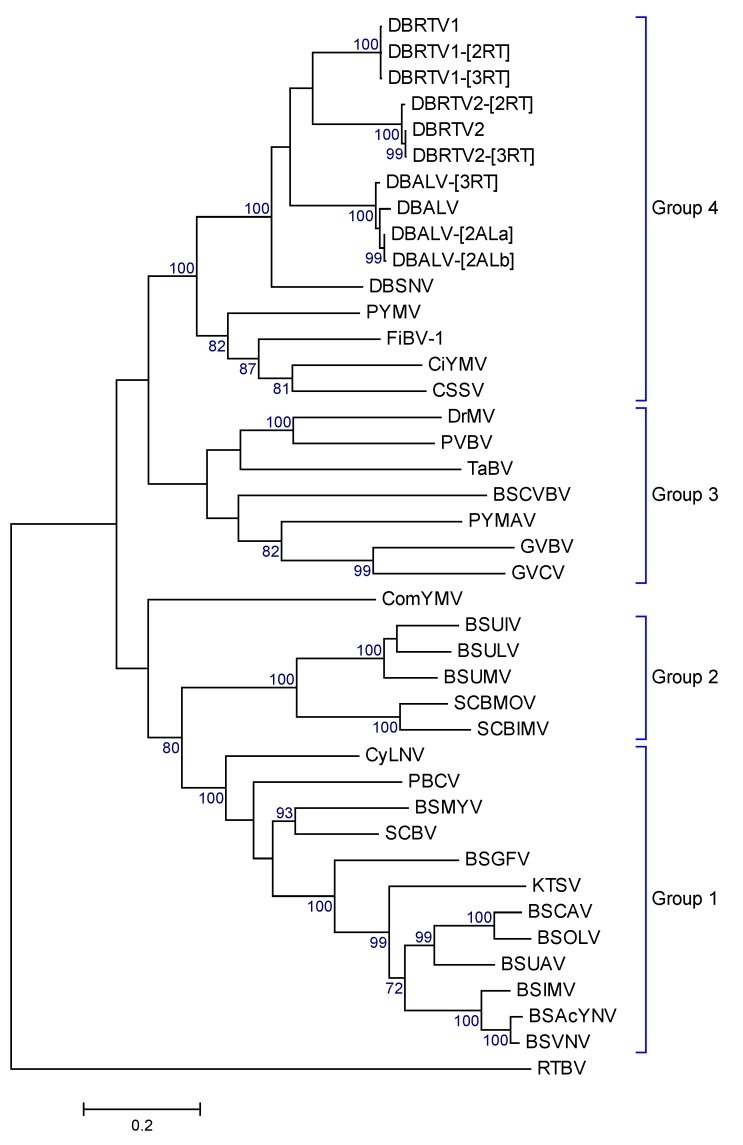
Maximum Likelihood phylogenetic tree obtained from the alignment of the deduced amino acid sequences of the ORF3 products of DBALV-[2ALa], DBALV-[2ALb], DBRTV1, DBRTV1-[2RT], DBRTV1-[2RT], DBRTV2, DBRTV2-[2RT], DBRTV2-[3RT], DBALV-[3RT], DBALV, DBSNV, other badnaviruses and related viruses (see text for detail). The phylogenetic tree was rooted to the polypeptide of RTBV. The topology of the tree supports the separation of the four major groups depicted by Wang et al. [58]. The bootstrap analysis of the sequences was 1000 replicates and the cut-off value was 70%. RTBV was used as outgroup.

Maximum Likelihood phylogenetic trees based on the complete genomic sequences, the ORF3 nucleotide sequences and the deduced amino acid sequences of ORF3 were constructed in order to understand the relationship between the nine viral genomes identified in this study, badnavirus genomes from other host plants and members of other genera within the family *Caulimoviridae*. The phylogenetic trees showed very comparable topologies for the complete genomic sequences, the ORF3 nucleotide sequences (Figure S4A,B) and the ORF3 amino acid sequences (Figure 6). All new nine yam badnavirus genomes identified in this study clustered together, and generated the same phylogenetic groups that have been described according to their partial RT-RNaseH sequences (Figure 2). The phylogenetic trees obtained also supported a closer phylogenetic relationship of the new yam badnavirus genomes to DBALV and DBSNV than to any badnavirus sequence from a different host plant (Figure 6 and Figure S4).

Taken together, all the results show that the nine virus genomes identified in this study are new Dioscorea bacilliform viruses and members of the genus *Badnavirus* within the family *Caulimoviridae*. We propose that the six sequences isolated from different *D. rotundata* accessions be recognised as two new species and be designated as Dioscorea bacilliform RT virus 1 (isolates DBRTV1 (KX008574), DBRTV1-[2RT] (KX008575), DBRTV1-[3RT] (KX008576)) and Dioscorea bacilliform RT virus 2 (isolates DBRTV2 (KX008577), DBRTV2-[2RT] (KX008578), DBRTV2-[3RT] (KX008579)). Two further sequences from *D. alata* and *D. rotundata* can be recognised as additional isolates of DBALV and be called DBALV-[2ALa/b] (a: KX008571, b: KX008572) and DBALV-[3RT] (KX008573), respectively.

## 4. Discussion

### 4.1. Potential of RCA/RFLP in Yam Badnavirus Diagnostics

In this study, the usefulness of the RCA/RFLP technique as a diagnostic tool to detect and obtain full-length episomal genomes of yam badnaviruses from yam total DNA samples was investigated. In this context, the potential of RCA as a method to facilitate the differentiation between episomal viral DNA from integrated sequences was also assessed. Using the sequence-independent multiply-primed RCA method resulted in the identification and characterisation of nine complete genome sequences of existing and previously undescribed yam badnavirus phylogenetic groups from different accessions of *D. alata* and *D. rotundata*. Moreover, the amplification of more than one badnavirus isolate from a single sample demonstrates the advantage of RCA for not only being sequence-independent but also its broad specificity through amplifying potentially all circular DNAs in a single reaction.

Several limitations to the usefulness of RCA for DBV diagnostic purposes were also uncovered in this study. We showed that the RCA/RFLP technique has a wider coverage of not only amplifying circular DNAs in a sample, but also linear templates at lower frequency. This led to the amplification of both episomal and also putatively integrated sequences as well as circular plant plastid sequences. A similar situation was reported in sweet potato [40] and in sugar beet [41], where plant mitochondrial DNAs were co-amplified during the desired detection of geminiviruses. The partial sequence NGl7-4Dr (KX008602) from *D. rotundata* TDr 1950B obtained here, was found to be a rearranged ORF3 (Figure 3), strongly suggesting that this sequence derived from an endogenous DBV. However, NGl7-4Dr could still be episomal, despite the fact that ORF3 is rearranged, as it may represent a defective-interfering sequence, whose replication is supported by the wild-type viral genome. A similar scenario was described by Umber et al. [30], where rearranged ORFs were obtained from RCA-generated integrated sequences in *D. rotundata*. Furthermore, analysis of a second clone from TDr 1950B (NGl1950Dr; KX008589) in this study identified a partial RT-RNaseH sequence that showed 100% nucleotide identity to endogenous sequence S1g6Dr (KF829974) [30]. This was unexpected, as RCA is not anticipated to amplify integrated sequences as these are not circular DNAs and the kinetics of the RCA reaction strongly favour circular templates [38,59]. Further analysis is needed to confirm if this sequence is directly from an endogenous sequence or from an episomal virus that has originated from an integrated sequence.

The amplification of plant DNA by RCA makes it unfavourable for rapid badnavirus indexing purposes as it increases time and labour costs by the need to discriminate between plant- and virus-derived RCA bands. Similarly, the appearance of multiple bands or faint bands in the restriction digestion analyses of RCA products hindered full result interpretation and led to inconclusive results. Hence using RCA as the only tool in DBV diagnostic studies, without the backup of an alternative diagnostic method, is not advisable as false-negative and false-positive results cannot be excluded. False-negative results could have important implications for quarantine measures, such as germplasm movement. In fact, a comparison of RCA with an RT-PCR approach specifically targeting episomal DBV transcripts indicates that false-negatives are rather common when employing RCA as a screening tool. Additionally, although a column purification step in the total nucleic extraction from yam was found to increase the efficiency of RCA, this will further increase the consumable cost of RCA, further limiting its potential as a diagnostic screening tool. In summary we come to the conclusion that RCA is useful for research purposes to characterise episomal viruses, but unsuitable for routine use in DBV diagnostics.

The post-amplification analysis of RCA products involves restriction digestion and the identification of a suitable restriction enzyme is of paramount importance to facilitate efficient detection of episomal yam badnavirus genomes. This is, however, only possible once the sequence of genomes to be screened for is known. The identification of a unique cutter is desirable, cutting yam badnavirus genomes at a single restriction site and resulting in full-length genomes of ~7–8 kbp which could then be cloned. Using a combination of SnapGene^®^ Viewer and the Restriction Analyzer (http://www.molbiotools.com/restrictionanalyzer.html), we identified *Cla*I (5′AT/CGAT-3′) as a unique cutter for all nine DBV genomes described in this study as well as DBSNV (DQ822073) and DBALV (DaBVb; X94575–X94582), but not DBALV (DaBVa; X94576–X94581). Although the strategy of using a unique cutter was applied successfully in the study performed by James et al. [33], such an approach is not likely to be universally applicable in either BSV or DBV diagnostics due to the considerable sequence variability known to exist within BSV and DBV isolates and hence the potentially high chance to miss unknown isolates [33]. Moreover, the use of a unique cutter in RCA product analyses for DBV detection may fail to reveal mixed infections, as the RCA products would result in restriction fragments of very similar sizes. Thus, the identification of a restriction enzyme that cuts more than once (for example a dual cutter) would be preferable. In this context, we identified *Xho*I (5′C/TCGAG-3′) as a suitable choice, cutting all DBV genomes at least once (DBRTV2-[2RT] and DBSNV), with the majority of the genomes having 2–3 restriction sites for this cutter and resulting in RFLP patterns that would be easily distinguishable and hence also clonable.

RCA-amplified viral DNAs can be tested for their infectivity [60]. Using unique cutters, simplified methods of constructing agro-infectious clones of begomovirus have been developed employing limited restriction enzyme digestion of RCA products [61,62]. The construction of an infectious DBV clone generated from RCA-amplified full-length genomes should be attempted in the future, to demonstrate infectivity of a yam badnavirus genome. This will be essential to verify whether the badnavirus genomes described in this study have all the hallmarks of replication-competent entities capable of inducing disease and existing as encapsidated forms in virions.

### 4.2. RCA-Captured Badnavirus Diversity

Phylogenetic analyses of the 22 partial RT-RNaseH sequences obtained from the RCA products clustered these sequences in five distinct phylogenetic groups. Although two of these groups (K05, and K08) had been previously identified [2], this study identified three new groups (T13, T14 and T15), increasing the total number of putative monophyletic yam badnavirus groups to 15. It is probable that many more undiscovered yam badnavirus species exist, particularly considering the high prevalence and diversity of yam badnaviruses recorded globally [2,8,9,11,12]. This study has increased the number of full-length yam badnavirus genome sequences from two (DBALV and DBSNV) to eleven, representing four species groups. An additional two of the episomal yam badnavirus groups identified by RCA here (K05 and T15) require further research to generate their full genomes. The episomal partial RT-RNaseH nucleotide sequences obtained from the RCA products exhibited diversity of up to 38% in this conserved region of the genus *Badnavirus*, confirming previous reports that badnaviruses are highly diverse, even within individual plants [8,25,26,27,28,29]. Eleven partial RT-RNaseH sequences that clustered in the K08 group demonstrated 75%–100% nucleotide identity to each other and 77%–96% with DBALV, the first yam badnavirus characterised. Bousalem et al. [9] described the DBV-A subgroup A (=K08) as more heterogeneous and displaying a higher average variability compared to DBV-A subgroup B, now classified as U12 [30]. Moreover, K08 was found to be polytomic but having significant structuring into subgroups, as exemplified by the subdivisions A1 and A2, described by Bousalem et al. [9]. According to this, DBALV-[3RT], NGb1892Dr2 and NGb2475Dr cluster within the DBV-A(A) A1 subgroup described by Bousalem et al. [9], whereas NGb0477Dr is part of the DBV-A(A) A2 subgroup.

The remaining sequences clustered into the other species groups K05, T13, T14 and T15 and had nucleotide identities in the range of 69%–73% with DBALV, 64%–77% with DBSNV and 69%–100% identity to each other. The cause of such large sequence diversity has not been determined, but as with other reverse transcribing viruses, it is likely to be due to mutations acquired during replication by reverse transcription [18,20].

Endogenous Dioscorea bacilliform virus (eDBV) sequences have been reported for species groups K05, K08, K09 and U12 [3,30]. Despite the clustering of an episomal sequence Gn155Dr (AM503383) in putative species group U12 [3], none of the 22 episomal partial RT-RNaseH sequences in this study clustered in this species group or K09. In contrast, two partial RT-RNaseH sequences (NGl1950Dr and NGb0477Dr) from RCA-amplified DNAs in this study did cluster with species groups K05 and K08, sharing 100% and 99% with known endogenous sequences of K05 (S1g6Dr, KF829974) and K08 (S2h9Dr, KF829997), respectively. This raises the question whether both sequences are derived from episomal (circular) or rather integrated (linear) DNAs, due to RCA also amplifying linear templates, as described above. The sequence NGb0477Dr also showed 99% nucleotide identity to BfA103Dc, Gn1633Dr, Gn845Dr and Gn502Dr (Figure 2). The sequence BfA103Dc (AM503393; [9]) was derived from a Pilimpikou yam sample which was reported as viral particle-free, whereas the other three sequences derived from yam samples of Guinea in which badnavirus particles were detected using immunosorbent electron microscopy (ISEM) [3]. It remains unclear, therefore, whether sequences NGl1950Dr and NGb0477Dr are derived from integrated sequences or episomal sequences thereof, and further research is in progress to elucidate this. Should the sequences be present in both episomal and integrated form, then this would suggest that these eDBV sequences represent more recent integration events that are still to be activatable in a similar manner to some eBSVs, where close to 100% nucleotide identity between an episomal BSV and an activatable eBSV has been described [63].

Some K08-RCA sequences obtained from *D. alata*, *D. cayenensis* and *D. rotundata* samples shared 91%–98% nucleotide identity with GenBank sequences from *D. bulbifera*, *D. alata* and *D. rotundata* that originated from different countries (Table 1). It was proposed previously that some badnaviruses of African origin have wide host range and are widespread in all yam-producing areas of the world [9]. The RCA results support episomal badnaviruses in group K08 being able to infect at least four *Dioscorea* species.

RCA resulted in the identification of several other partial sequences, but these were not characterised further due to their high nucleotide identity with either DBALV or DBSNV. One of the partial sequences, DBSNV2 (~4.3 kbp), obtained from *D. cayenensis* TDc 3709B shared 98.1% nucleotide identity with DBSNV (DQ822073). Therefore, the isolate can be considered as another sequence of DBSNV, but this time detected in *D. cayenensis*. This result supports the proposition by Seal and Muller [6] that there are possibilities of viral transmission between cultivated and wild yam species such as *D. sansibarensis*, which are often grown on the edges of cultivated yam-fields for the security of cultivated species from thieves [64].

### 4.3. Full-Length Sequences of Dioscorea Bacilliform Viruses

The characterisation of novel yam badnaviruses will assist in improving yam badnavirus diagnostics and possibly help in understanding DBV integration events by the comparison of highly similar sequences of both episomal and endogenous nature. The nine new full-length genomes of yam badnavirus isolates identified in this study originated from *D. rotundata* (TDr 89/02475, TDr 1892 and Adaka) and *D. alata* (TDa 85/00250). Sequence analyses of these nine genome sequences confirm that they are members of the genus *Badnavirus.* The ORFs of DBALV [4] and DBSNV [6] were reported to contain three ORFs, a P1 protein of ~16.8 kDa of unknown function, the virion-associated protein P2 of ~14 kDa, and the polyprotein P3 of ~214 kDa [17]. The genome sequences described in this study display three ORFs similar to these yam badnaviruses (Figure S3 and Table S2). Although the functions of the gene products encoded by ORFs 1 and 2 are poorly understood, it has been postulated that the protein of ORF1 associates the virus with plant cell components and may be involved in mealybug transmission [65,66]. ORF2 protein has been proposed to be involved in virus assembly because of its nucleic acid-binding activity [67]. Furthermore, Leclerc et al. [68] discovered a conserved coiled-coil motif present at the N-terminus of the ORF3 product of all members of the genus *Caulimovirus* and within ORF2 of badnaviruses and RTBV. The proteins were called VAPs (virion-associated proteins) because of the association with the capsid protein in the virion shells of cauliflower mosaic virus (CaMV) [69], which in the case of CaMV were shown to assemble as tetramers and could act as the “arm” of the virus particle by keeping its C-terminus anchored into the capsid shell and exposing the tetramer for interaction with other proteins [70]. The putative coiled-coil domain identified in ORF2 of DBALV depicted by Stavalone et al. [70] appears conserved in all new yam badnavirus genomes described in this study and can be located at position 48–71 of the protein alignment (Figure S1B). The aforementioned function of the VAP proteins potentially makes ORF2 a suitable target for the design of specific yam badnavirus antisera.

In summary, the comparative analyses of the protein sequences encoded by ORF1 and ORF2 of the nine isolates, together with the equivalent sequences for DBALV and DBSNV, revealed a high degree of conserved amino acid sequences. Interestingly, the results also suggested group-specific amino acid sequence patterns, in particular in ORF2 (Figure S1A,B). The identification of the putative coat proteins in the new badnavirus isolates described in this study, together with the coat protein sequences of DBALV and DBSNV (Figure S2), could, when compared to other badnaviruses (Figure 5), contribute to the design of a generic yam badnavirus-specific antisera much needed by yam breeding and multiplication programmes [2,3].

Phylogenetic analyses of the complete genome sequences and the nucleotide as well as deduced amino acid sequences of ORF3 of DBRTV1, DBRTV1-[2RT], DBRTV1-[3RT]), DBRTV2, DBRTV2-[2RT], DBRTV2-[3RT]), DBALV-[3RT] and DBALV-[2ALa/b] genomes revealed a closer phylogenetic relationship of all yam badnaviruses to each other than to any other known badnavirus, which is in agreement with previous reports [6]. The phylogenetic trees, similar to those generated by Wang et al. [58], showed very comparable topologies (Figure 6 and Figure S4), supporting the separation of the ComYMV and CSSV subgroups in the genus *Badnavirus* described by Xu et al. [55]. The topologies of the trees also support the separation of the four major groups depicted by Wang et al. [58], where group one (which, for example, contains the majority of BSV isolates) and two together and group three and four together resemble the ComYMV and CSSV subgroups with further separations, respectively. These relationships suggest that members of the genus *Badnavirus* are more closely related according to plant host family than geographical origin, presumably due to host specificity and past exchange of infected germplasm.

## 5. Conclusions

In summary, a combination of RCA and PCR methods for the amplification and characterisation of new complete genomic yam badnavirus sequences corresponding to existing and previously undescribed yam badnavirus species are described here for the first time. This approach resulted in the identification of a total of nine new full-length yam badnavirus genomes, and we propose that six sequences isolated from different *D. rotundata* accession be recognised as two new species and be designated as Dioscorea bacilliform RT virus 1 (isolates DBRTV1 (KX008574), DBRTV1-[2RT] (KX008575), DBRTV1-[3RT] (KX008576)) and Dioscorea bacilliform RT virus 2 (isolates DBRTV2 (KX008577), DBRTV2-[2RT] (KX008578), DBRTV2-[3RT] (KX008579)). Two further sequences from *D. alata* and *D. rotundata* can be recognised as additional isolates of DBALV and be called DBALV-[2ALa/b] (a: KX008571, b: KX008572) and DBALV-[3RT] (KX008573), respectively.

We set out to assess the potential of RCA as a method to facilitate the differentiation between episomal viral DNA from integrated sequences in DBV diagnostics. Our results provide experimental evidence that RCA is capable of targeting linear templates as well as circular episomal sequences. Also, our experience using RCA shows that the chance for false-negative results is relatively high and thus of great concern when using RCA for indexing of yam breeding lines without the parallel use of an additional diagnostic method. RCA is hence more useful for research purposes than for DBV diagnostic purposes.

The results presented in this study provided direct experimental evidence for the presence of badnavirus infections in West African yam germplasm, confirming findings by Seal et al. [3], which emphasized the lack of virus-free planting material as being a major constraint to improvement of yam yields in this region [71]. In fact, our results showed that mixed badnavirus infections occur relatively often, as we found in six of the 12 yam lines analysed in detail. Phylogenetic analyses of RCA-derived partial RT-RNaseH sequences obtained here clustered 22 sequences in five distinct phylogenetic groups. Although two of these groups (K05, and K08) had been previously described [2], this study identified three new groups (T13, T14 and T15), increasing the total number of putative monophyletic groups to 15. Also, our findings have increased the number of full-length yam badnavirus genome sequences from two (DBALV and DBSNV) to eleven, representing four species groups. However, two of the episomal yam badnavirus groups identified by RCA here (K05 and T15) still await full genome sequence characterisation and it is probable that many more species of infectious yam badnaviruses exist. Only future investigations will be able to assess to what extent the obtained sequence data of complete viral genomes represent infectious entities. Sequence data alone cannot cover information needed to fulfil Koch’s postulates [72], comprising infectivity and transmission. Proof of infectivity is not only required for taxonomic recognition but seminal to address further questions regarding epidemiology as well as evolution of these viruses in order to secure healthy yam germplasm and food production.

## Figures and Tables

**Table 1:** RCA-derived sequences and BLAST analysis of their partial RT-RNaseH (528 bp) coding regions.

Plant Accession ^a^	RCA Sequence	Accession	Size (bp)	NCBI Nearest Match	Identity (%)	Species Group ^e^
Adaka (TDr)	DBRTV2-[2RT] ^d^	KX008578	7462	S1un5Dr (KF830000)	72	T14
TDa 00/00005	NGb0005Da1 ^b^	KX008580	528	NG1Da (AM944571)	96	K08
	NGb0005Da2 ^b^	KX008581	528	S1g6Dr (KF829974)	92	K05
TDa 85/00250	DBALV-[2ALa]	KX008571	7544	VU249Db (AM072705)	94	K08
	DBALV-[2ALb]	KX008572	7544	VU249Db (AM072705)	94	K08
TDa 95/00310	NGb0310Da1 ^b^	KX008582	528	NG1Da (AM944571)	98	K08
	NGb0310Da2 ^b^	KX008583	528	NG3Da (AM944573)	97	K08
	NGb0310Da3 ^b^	KX008584	528	TG2Dr (AM944580)	90	T15
TDc 3841A	NGl3841Dc ^b^	KX008585	528	FJ60bDr (AM072659)	91	K05
TDr 04/00219 × TDr 97/00777	NGb0477Dr ^b^	KX008586	528	BfA103Dc (AM503393)	99	K08
TDr 1892	DBRTV2 ^d^	KX008577	7438	FJ60bDr (AM072659)	72	T14
	NGb1892Dr1 ^b^	KX008587	528	NG1Da (AM944571)	88	K08
	NGb1892Dr2 ^b^	KX008588	528	NG01Dr (AM072673)	86	K08
TDr 1892B	DBRTV1-[2RT] ^c^	KX008575	7707	S2f8Dr (KF829993)	77	T13
	DBRTV2-[3RT] ^d^	KX008579	7438	FJ60bDr (AM072659)	72	T14
	DBALV-[3RT] ^c^	KX008573	7609	NG01Dr (AM072673)	99	K08
TDr 1950B	NGl1950Dr ^b^	KX008589	528	S1g6Dr (KF829974)	100	K05
TDr 89/02475	DBRTV1	KX008574	7702	S2f8Dr (KF829993)	76	T13
	NGb2475Dr ^b^	KX008590	528	NG01Dr (AM072673)	86	K08
TDr 89/02475A	DBRTV1-[3RT] ^d^	KX008576	7708	S2f8Dr (KF829993)	76	T13
TDr 95/18544	NGb1844Dr1 ^b^	KX008591	528	S2f8Dr (KF829993)	76	T13
	NGb1844Dr2 ^b^	KX008592	528	VU249Db (AM072705)	94	K08

^a^ The host plants are represented by plant accession, TDa = *D. alata*, TDc = *D. cayenensis* and TDr = *D. rotundata*; ^b^ Sequences derived from PCR amplifications using Badna-FP/-RP primer pair with RCA fragments as template; ^c^ Combination of RCA and PCR clones; ^d^ Derived from PCR clones; ^e^ According to phylogenetic tree (Figure 2).

## Supplementary Materials

The following are available online at www.mdpi.com/1999-4915/8/7/188/s1, Figure S1: Protein alignment from deduced amino acid sequences of the ORF1 (A) and ORF2 (B) products of DBALV-[2ALa], DBALV-[2ALb], DBRTV1, DBRTV1-[2RT], DBRTV1-[2RT], DBRTV2, DBRTV2-[2RT], DBRTV2-[3RT], DBALV-[3RT], DBALV, DBSNV, Figure S2: Comparison of highly conserved amino acid residues in the coat protein encoded by the N-terminal half of the ORF 3 product among DBALV-[2ALa], DBALV-[2ALb], DBRTV1, DBRTV1-[2RT], DBRTV1-[2RT], DBRTV2, DBRTV2-[2RT], DBRTV2-[3RT], DBALV-[3RT], DBALV, DBSNV separately and together with 14 badnavirus and other members of the family *Caulimoviridae*, Figure S3: Comparative linear representation of DBALV, DBALV-[2AL], DBALV-[3RT], DBSNV, DBRTV1 and DBRTV2 genome, Figure S4: Maximum Likelihood phylogenetic trees obtained from alignments of the complete genomic sequences and the nucleotide sequences of the ORF3 products of DBALV-[2ALa], DBALV-[2ALb], DBRTV1, DBRTV1-[2RT], DBRTV1-[2RT], DBRTV2, DBRTV2-[2RT], DBRTV2-[3RT], DBALV-[3RT], DBALV, DBSNV, other badnaviruses and related viruses, Table S1: Primers used to clone DBRTV1, DBRTV2 and DBALV-[3RT] genomes, Table S2: Comparison of genome features of eleven yam badnavirus genomes.

## Abbreviations

The following abbreviations are used in this manuscript:

aa	amino acid
BLAST	basic local alignment search tool
BSV	banana streak virus
CP	capsid protein
CTAB	cetyltrimethylammonium bromide
DBALV	*Dioscorea bacilliform alata virus*
DBRTV	Dioscorea bacilliform rotundata (RT) virus
DBSNV	*Dioscorea bacilliform sansibarensis virus*
DBV	Dioscorea bacilliform virus
DOAJ	Directory of open access journals
ds	double stranded
eDBVs	endogenous Dioscorea bacilliform viruses
EPRV	endogenous pararetrovirus
IC-PCR	immunocapture-PCR
ICTV	International Committee on Taxonomy of Viruses
IG	intergenic region
ISEM	immunosorbent electron microscopy
kbp	kilo base pairs
LD	linear dichroism
MAFFT	Multiple Alignment using Fast Fourier Transform
MDPI	Multidisciplinary Digital Publishing Institute
MP	movement protein
NCBI	National Centre for Biotechnology Information
ORF	open reading frame
PCR	polymerase chain reaction
PR	pepsin-like aspartate protease
RCA	rolling circle amplification
RFLP	restriction fragment length polymorphism
RNaseH	ribonuclease H
RT	reverse transcriptase
RT-PCR	reverse transcription-PCR
SDW	sterile distilled deionised water
SSA	Sub-Saharan Africa
TDa	*Dioscorea alata* accession
TDc	*Dioscorea cayenensis* accession
TDr	*Dioscorea rotundata* accession
TLA	Three letter acronym
VAP	virion-associated protein
Zn knuckle	zinc-finger domain
