# Supplementary material for: A Sequence-Independent Strategy for Amplification and Characterisation of Episomal Badnavirus Sequences Reveals Three Previously Uncharacterised Yam Badnaviruses
Source: Viruses. 2016 Jul 7;8(7):188. doi: 10.3390/v8070188 (PMC4974523; doi:10.3390/v8070188)
Supplement: Supplementary file 1 [file viruses-08-00188-s001.pdf]

Supplementary Materials: A Sequence-Independent Strategy for Amplification and Characterisation of Episomal Badnavirus Sequences Reveals Three Previously Uncharacterised Yam Badnaviruses

Moritz Bömer, Aliyu A. Turaki, Gonçalo Silva, P. Lava Kumar and Susan E. Seal

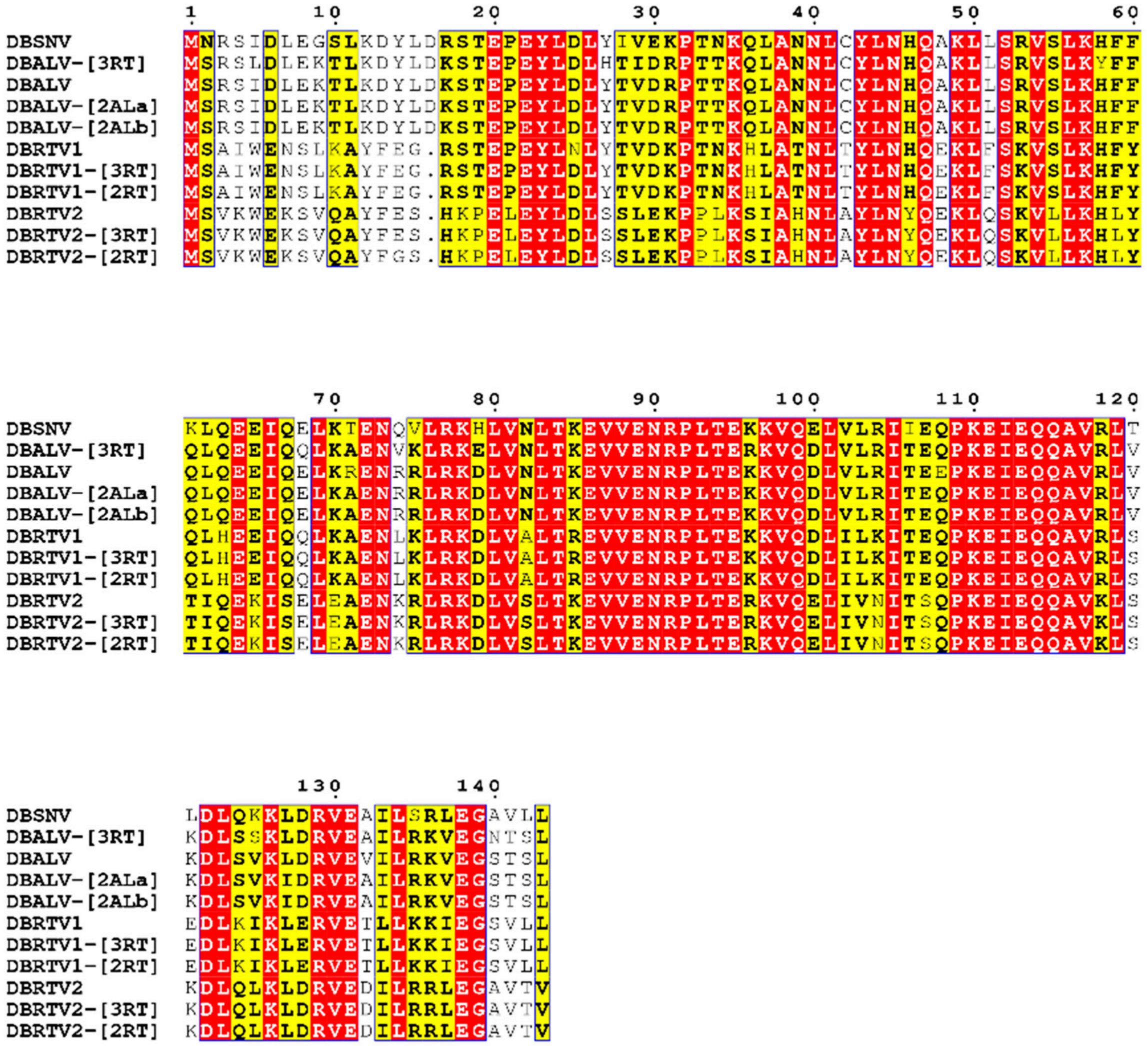

(A)

Figure S1. *Cont.*

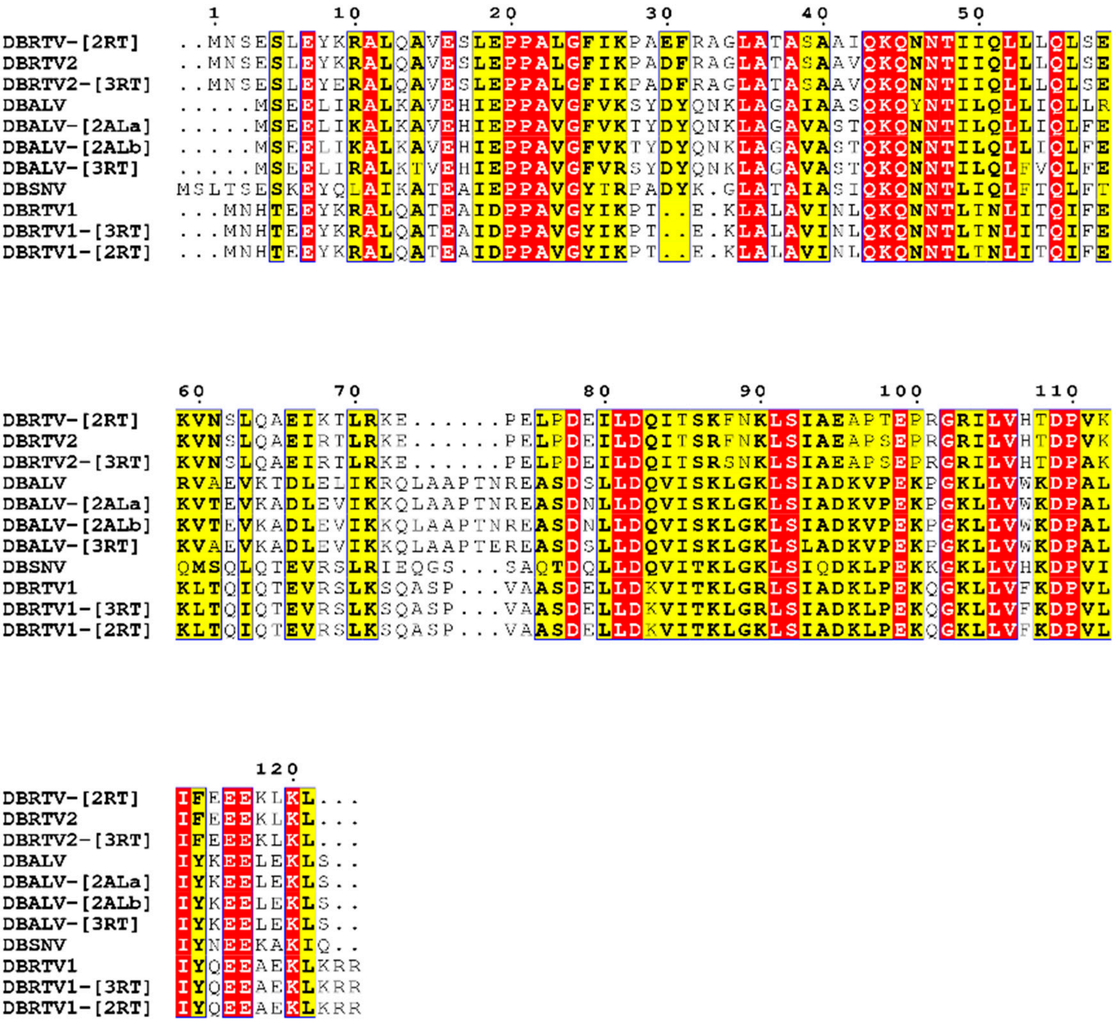

(**B**)

Figure S1. Protein alignment from deduced amino acid sequences of the ORF1 (**A**) and ORF2 (**B**) products of DBALV-[2ALa], DBALV-[2ALb], DBRTV1, DBRTV1-[2RT], DBRTV1-[2RT], DBRTV2, DBRTV2-[2RT], DBRTV2-[3RT], DBALV-[3RT], DBALV, DBSNV. Sequences were aligned with CLUSTAL OMEGA (http://www.ebi.ac.uk/Tools/msa/clustalo/) and coloured using ESPript 3.0 [1], where functional conserved amino acid residues are highlighted with yellow backgrounds and complete consistent residues with red backgrounds.

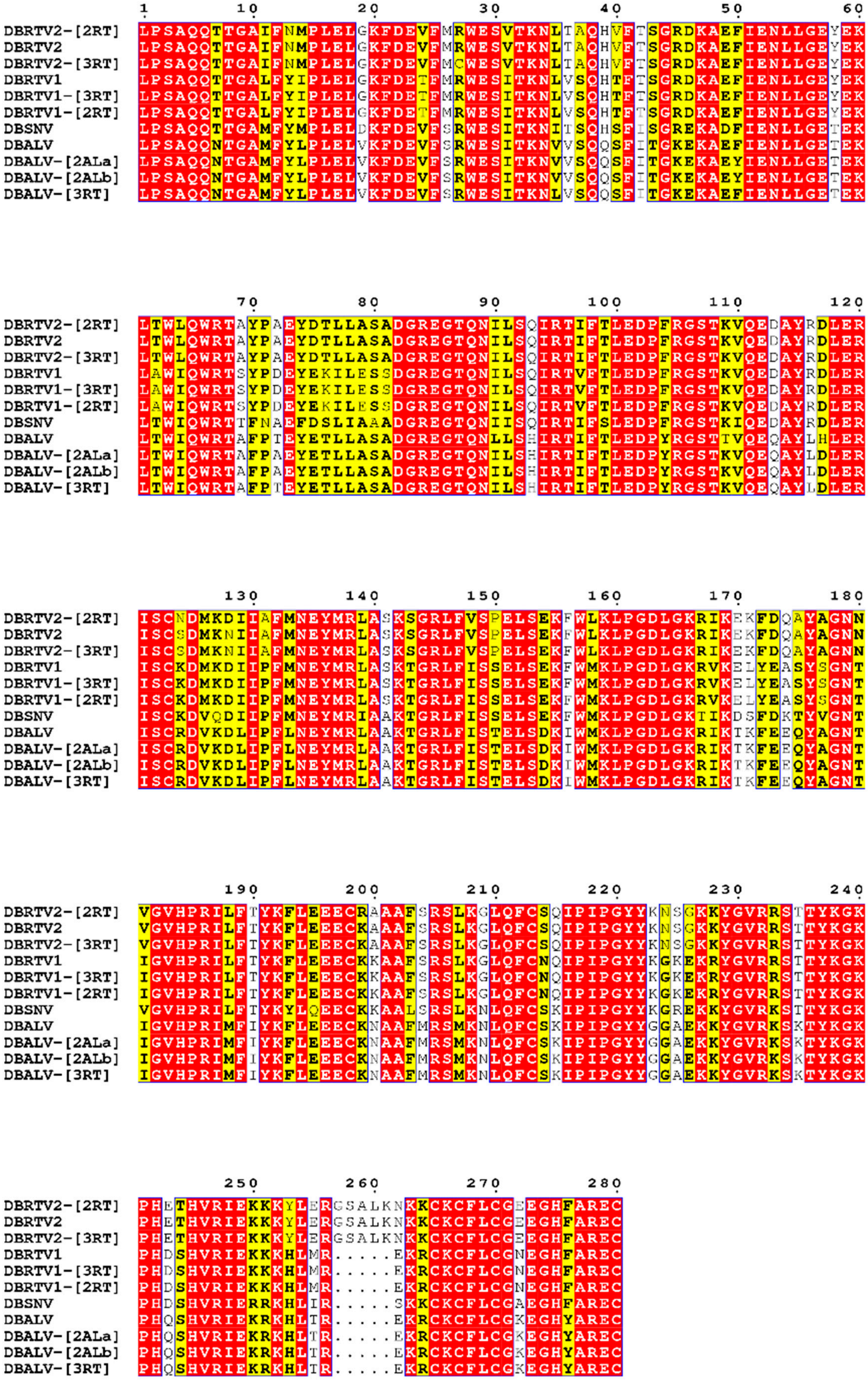

(**A**)

Figure S2. *Cont.*

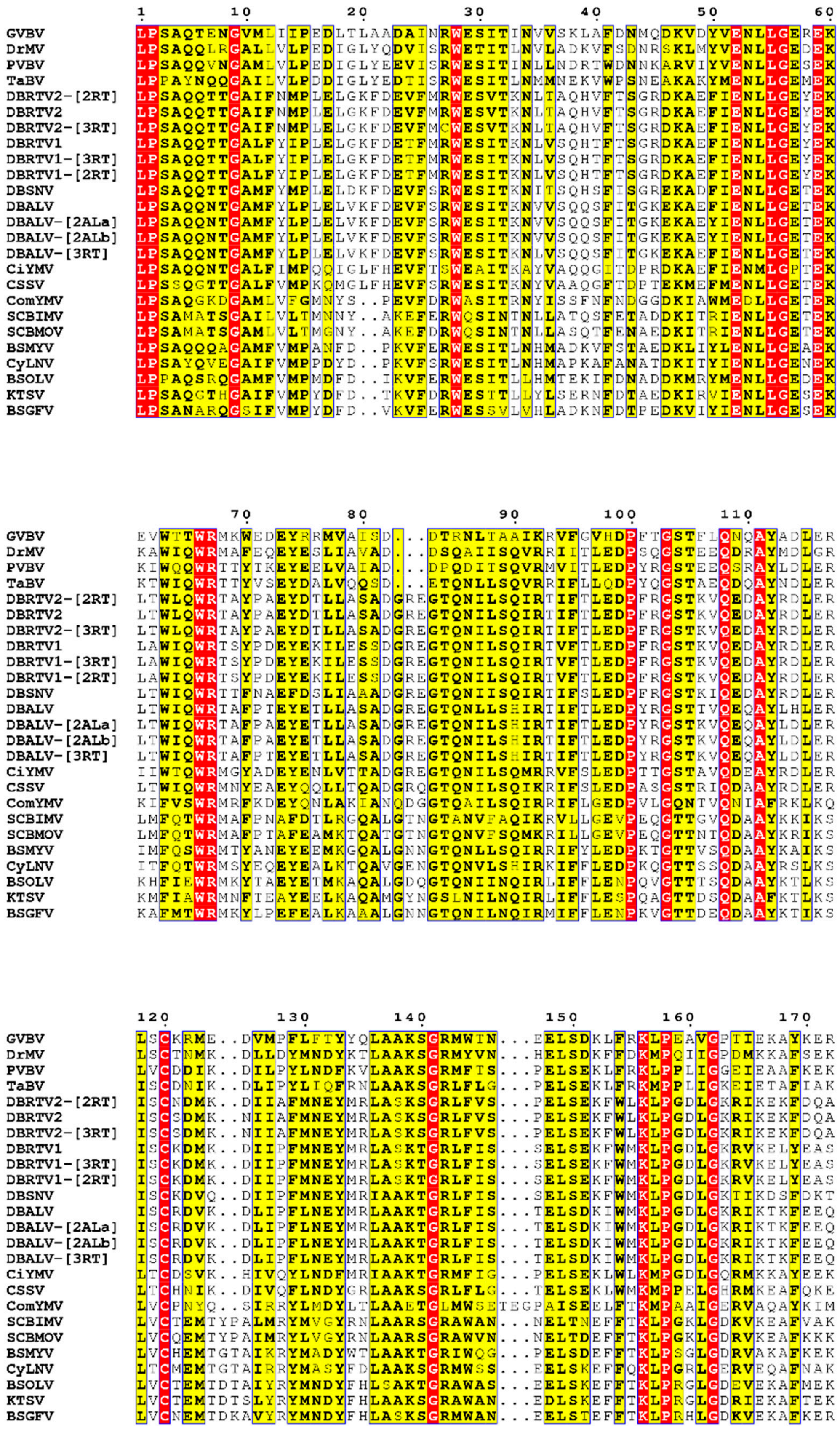

Figure S2. *Cont.*

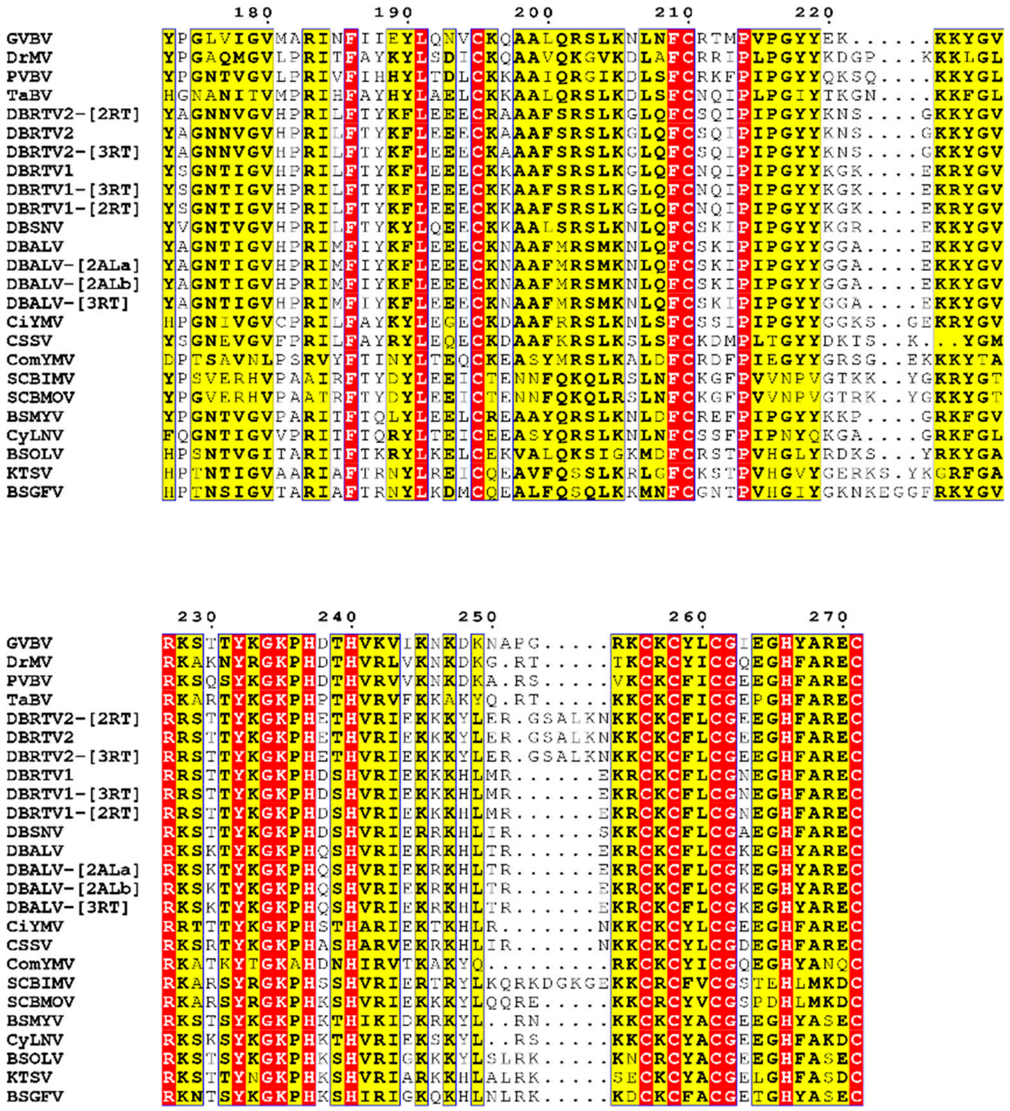

(**B**)

Figure S2. Comparison of highly conserved amino acid residues in the coat protein encoded by the N-terminal half of the ORF 3 product among DBALV-[2ALa], DBALV-[2ALb], DBRTV1, DBRTV1-[2RT], DBRTV1-[2RT], DBRTV2, DBRTV2-[2RT], DBRTV2-[3RT], DBALV-[3RT], DBALV, DBSNV separately (**A**) and together with 14 badnavirus and other members of the family Caulimoviridae (see text for detail) (**B**). Sequences were aligned with CLUSTAL OMEGA (http://www.ebi.ac.uk/Tools/msa/clustalo/) and coloured using ESPript 3.0 [1], where functional conserved amino acid residues are highlighted with yellow backgrounds and complete consistent residues with red backgrounds.

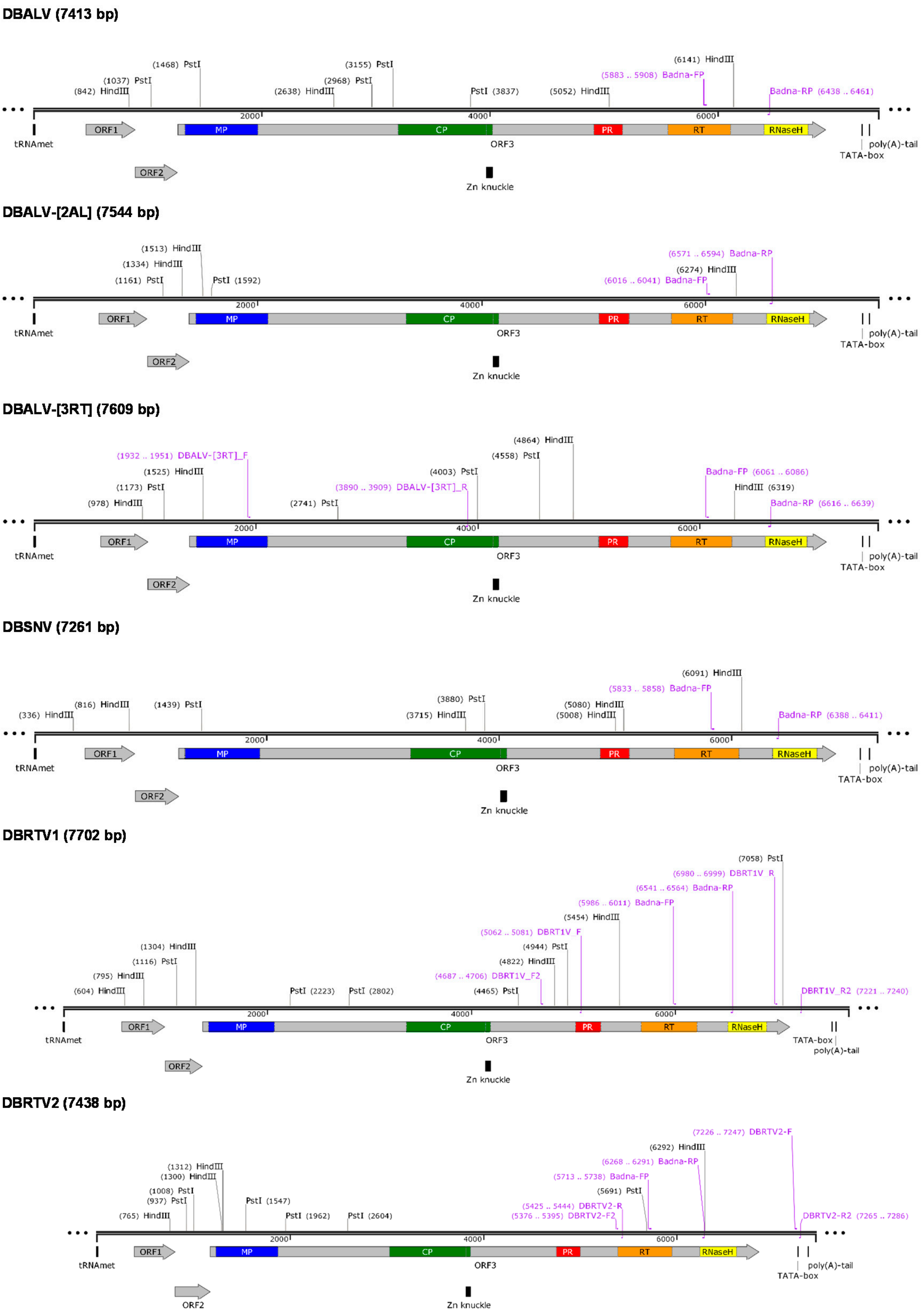

Figure S3. Comparative linear representation of DBALV, DBALV-[2AL], DBALV-[3RT], DBSNV, DBRTV1 and DBRTV2 genome showing tRNAMet-binding site; the TATA-box; the putative poly(A)-tail; open reading frame (ORF)1; ORF2; ORF3 with putative movement protein (MP), capsid protein zinc-finger domain (CP and Zn Knuckle), pepsin-like aspartate protease (PR), reverse transcriptase (RT) and RNaseH (RNaseH) motifs; the exact positions of the Badna F/-R primers; the DBRTV1, DBRTV2 and DBALV-[3RT] specific primers for PCR amplification of the complete genome and the restriction sites for PstI and HindIII.

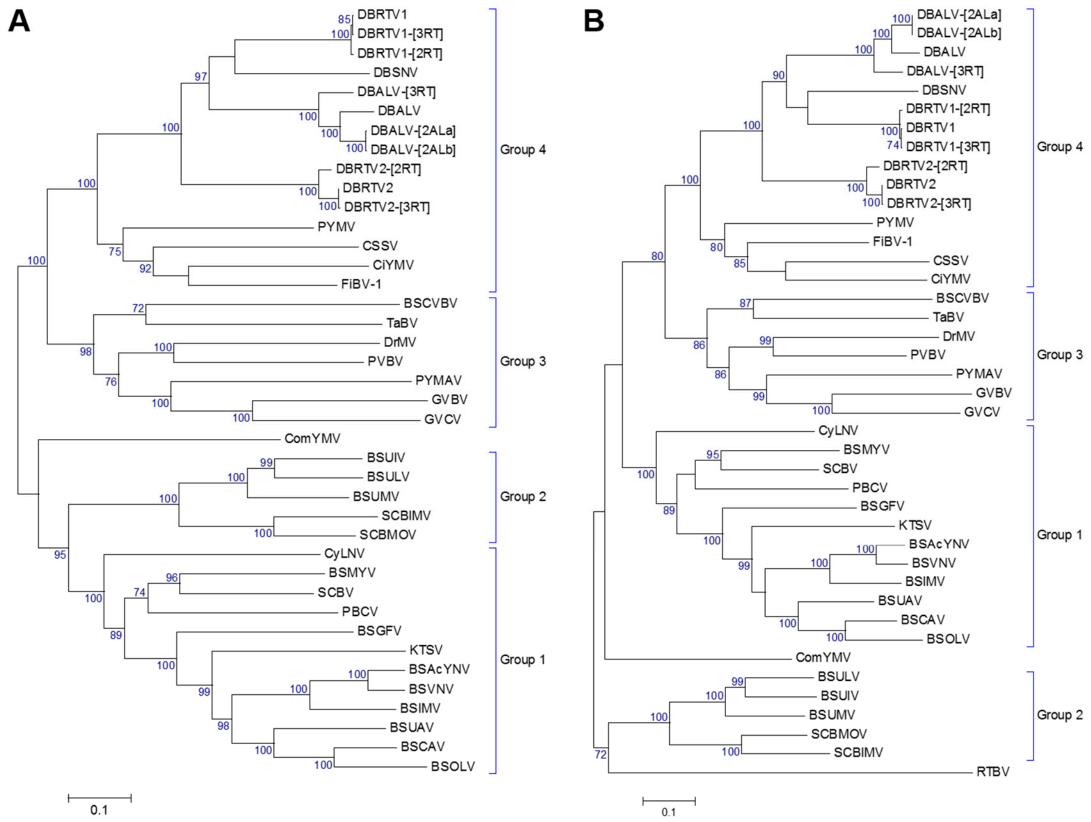

Figure S4. Maximum Likelihood phylogenetic trees obtained from alignments of the complete genomic sequences (**A**) and the nucleotide sequences of the ORF3 products (**B**) of DBALV-[2ALa], DBALV-[2ALb], DBRTV1, DBRTV1-[2RT], DBRTV1-[2RT], DBRTV2, DBRTV2-[2RT], DBRTV2-[3RT], DBALV-[3RT], DBALV, DBSNV, other badnaviruses and related viruses (see text for detail); The phylogenetic tree of the ORF3 nucleotide sequences was rooted to the nucleotide sequence of the polypeptide of RTBV (**B**). The topologies of the trees support the separation of the four major groups depicted by Wang et al. [2]. The bootstrap analysis of the sequences was 1000 replicates and the cut-off value was 70%.

References

1. Robert, X.; Gouet, P. Deciphering key features in protein structures with the new ENDscript server. *Nucleic Acids Res.* **2014**, *42*, 320–324.
2. Wang, Y.; Cheng, X.; Wu, X.; Wang, A.; Wu, X. Characterization of complete genome and small RNA profile of pagoda yellow mosaic associated virus, a novel badnavirus in China. *Virus Res.* **2014**, *188*, 103–108.

© 2016 by the authors. Submitted for possible open access publication under the terms and conditions of the Creative Commons Attribution (CC-BY) license (http://creativecommons.org/licenses/by/4.0/).
